# *Clostridiodes difficile* in COVID-19 Patients, Detroit, Michigan, USA, March–April 2020

**DOI:** 10.3201/eid2609.202505

**Published:** 2020-09

**Authors:** Saraswathi Lakkasani, Kok Hoe Chan, Hamid S. Shaaban

**Affiliations:** Saint Michael’s Medical Center, New York Medical College, Newark, New Jersey, USA

**Keywords:** COVID-19, SARS-CoV-2, innate immunity, IL-12, IFN-γ, Clostridiodes difficile, bacteria, viruses, respiratory infections, severe acute respiratory syndrome coronavirus 2, 2019 novel coronavirus disease, coronavirus disease, zoonoses, coronavirus, gastrointestinal symptoms, enteric infections

**To the Editor:** Sandhu et al. (*1*) reported 9 patients who were co-infected with severe acute respiratory syndrome coronavirus 2 (SARS-CoV-2) and *Clostridioides difficile*. *C. difficile* infection (CDI) can be a co-occurrence or result of antimicrobial drug overuse and is potentially a complication of coronavirus disease (COVID-19). We report a 52-year-old man with hypertension who had fever, respiratory symptoms, abdominal pain, and diarrhea for 3 days. At admission to Saint Michael’s Medical Center (Newark, New Jersey, USA), he had a temperature of 101.8°F but was otherwise hemodynamically stable. He had an elevated absolute lymphocyte count (700 cells/μL), indicating lymphopenia. He tested positive for SARS-CoV-2 RNA by reverse transcription PCR and had elevated inflammatory markers on blood profile. He tested positive for *C. difficile* toxin and antigen at admission. He did not use antimicrobial drugs or proton pump inhibitors and had no known contacts with persons with diarrhea. He was mechanically ventilated and received oral vancomycin, intravenous metronidazole, and vasopressors. He died of respiratory failure and septic shock. In comparison to the patients described by Sandhu et al., the patient we report was younger and did not have a history of antimicrobial use.

SARS-CoV-2 has multifaceted presentations. Angiotensin-converting enzyme 2 receptor, which can act as a receptor for severe acute respiratory syndrome coronavirus, is expressed not only in alveolar cells but also in the gastrointestinal tract, including colonic cells (*2*,*3*). Diarrhea associated with COVID-19 might erode the normal microbial flora of the gut, leading to increased risk for CDI. Also, COVID-19 might weaken the immune system, leaving the patient vulnerable to CDI. COVID-19 patients produce inadequate interferon-γ and have defective macrophage activation and function, resulting in a dysregulated immune response (*4*). Interleukin-12 and interferon-γ are components of cell-mediated immunity. Interferon-γ produced by T-helper cells induces macrophages to destroy bacteria such as *C. difficile* (*5*). 

The relationship between SARS-CoV-2 and CDI is still poorly understood. CDI might be a complication of COVID-19; however, we could not exclude the possibility of co-occurrence of CDI with COVID-19. Physicians should consider CDI when encountering a COVID-19 patient with diarrhea.
